# Comparison of Surface-Bound and Free-Standing Variations of HKUST-1 MOFs: Effect of Activation and Ammonia Exposure on Morphology, Crystallinity, and Composition

**DOI:** 10.3390/nano8090650

**Published:** 2018-08-23

**Authors:** Brandon H. Bowser, Landon J. Brower, Monica L. Ohnsorg, Lauren K. Gentry, Christopher K. Beaudoin, Mary E. Anderson

**Affiliations:** Department of Chemistry, Hope College, 35 E. 12th Street, Holland, MI 49422, USA; bhbowser@gmail.com (B.H.B.); landon.brower@hope.edu (L.J.B.); monica.ohnsorg@gmail.com (M.L.O.); Lauren.gentry@memphistr.org (L.K.G.); chrbeaudoin@gmail.com (C.K.B.)

**Keywords:** metal-organic framework, microscopy, thin films, powders

## Abstract

Metal-organic frameworks (MOFs) are extremely porous, crystalline materials with high surface area for potential use in gas storage, sequestration, and separations. Toward incorporation into structures for these applications, this study compares three variations of surface-bound and free-standing HKUST-1 MOF structures: surface-anchored MOF (surMOF) thin film, drop-cast film, and bulk powder. Herein, effects of HKUST-1 ammonia interaction and framework activation, which is removal of guest molecules via heat, are investigated. Impact on morphology and crystal structure as a function of surface confinement and size variance are examined. Scanning probe microscopy, scanning electron microscopy, powder X-ray diffraction, Fourier-transform infrared spectroscopy, and energy dispersive X-ray spectroscopy monitor changes in morphology and crystal structure, track ammonia uptake, and examine elemental composition. After fabrication, ammonia uptake is observed for all MOF variations, but reveals dramatic morphological and crystal structure changes. However, activation of the framework was found to stabilize morphology. For activated surMOF films, findings demonstrate consistent morphology throughout uptake, removal, and recycling of ammonia over multiple exposures. To understand morphological effects, additional ammonia exposure experiments with controlled post-synthetic solvent adsorbates were conducted utilizing a HKUST-1 standard powder. These findings are foundational for determining the capabilities and limitation of MOF films and powders.

## 1. Introduction

Highly porous crystalline materials known as metal-organic frameworks (MOFs) have been the focus of much attention over recent years due to their promising potential for a wide range of applications, including gas storage, separation, catalysis, and sensing [1,2,3]. Careful selection of the inorganic nodes and organic linkers that compose the chemical structure of the material can lead to a variety of highly tunable pore sizes and chemical functionality [4]. Varying synthetic conditions, such as processing solvent [5], temperature [6], concentration [7], etc., also allows one to tailor MOF properties. This careful control makes it possible to embed MOF materials within an array of hierarchical architectures and composites [8]. Once synthesized, MOF crystallinity and morphology are typically evaluated to determine quality. However, when testing the materials in the presence of different gases, it is rare to find studies that monitor possible morphological changes, even though changes in crystal structure are often investigated [9]. This lack of structural understanding needs to be addressed for a variety of MOF systems and host-guest interactions if they are to become a viable option for integration into more complex designs. This study herein characterizes the morphological impact of one such interaction for the HKUST-1 MOF, providing previously neglected insights into how the MOF may perform in modern applications.

**Figure 1:**
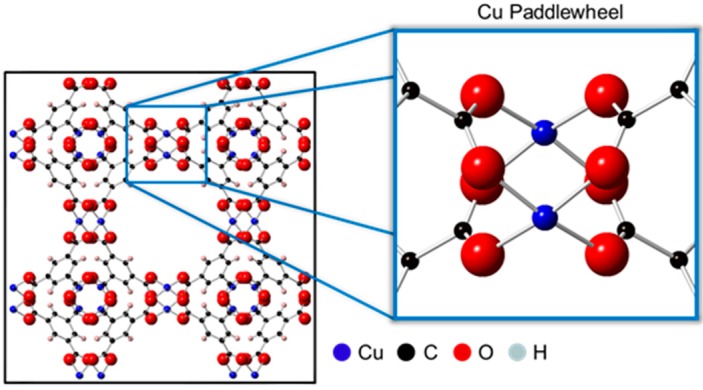
Unit cell (left) of the HKUST-1 metal-organic framework system ((100) crystal face) [10]. HKUST-1 is composed of four benzene-1,3,5-tricarboxylate organic ligands coordinated to Cu^2+^ dimer. Highlighted is the copper paddlewheel structure (right) characteristic of the HKUST-1 system. Each Cu^2+^ dimer completes its octahedral coordination sphere with two axial positions (vacant here) opposite of the Cu-Cu vector.

The most widely studied MOF is HKUST-1, also known as Cu_3_BTC_2_ or MOF-199, which was first discovered by Chui et al. [10] and uses copper(II) ions and benzene-1,3,5-tricarboxylate (BTC) as the inorganic and organic building blocks, respectively. One of the reasons HKUST-1 has garnered so much attention is because of its ability to capture and store a wide variety of gases, including carbon monoxide, carbon dioxide, nitric oxide, nitrogen, hydrogen, and sulfur dioxide [11,12,13]. Furthermore, HKUST-1 has been shown to be a suitable candidate for the sequestration of harmful gases like hydrogen sulfide, arsine, and ammonia [14,15]. One of the key features of HKUST-1 is that it allows for the adsorption of these different gases in the copper paddlewheel unit, depicted in Figure 1. In this paddlewheel unit, Cu^2+^ ions are connected to form Cu^2+^ dimers through a weak bond and are bridged by four carboxylate units. The as-synthesized framework typically contains solvent molecules weakly bound to the axial coordination sites of the Cu^2+^ ions. These solvent molecules can be removed through a simple activation procedure (i.e., heating under vacuum), thus enabling the Cu^2+^ ions to act as Lewis acidic sites for the binding of molecules possessing basic character, such as ammonia. Herein, this research explores the effect of residual solvent molecules on the adsorption of ammonia in HKUST-1, highlighting for the first time how the presence of solvent (or lack thereof) affects morphology. 

The synthesis of HKUST-1 can be varied so as to achieve a wide range of geometries, morphologies, and composites, allowing the MOF to be tailored toward specific applications [16]. For example, HKUST-1 can be tethered to a substrate as a surface-anchored MOF (surMOF) or a drop-cast thin film; and it can be synthesized to be a free-standing material (bulk powder). However, most studies for HKUST-1 only characterize and analyze one of its possible variations, typically its powder form. This is especially true in the investigation of the HKUST-1 interaction with ammonia gas. There have been studies that compare pure HKUST-1 powder to composite materials that contain it, but there is a deficiency of ammonia studies that directly compare different MOF variations [17]. 

The research herein compares the behavior of surface-bound and free-standing HKUST-1 materials. A study by Nijem et al. [18] suggested that aspects of the HKUST-1 interaction with ammonia may vary depending on whether the MOF is confined to a substrate. Also, the effect of size on MOF behavior is studied; the three MOF variations investigated herein all had the same chemical composition but were on different size scales. The aim of the study is to investigate how these different variations respond to ammonia in order to gain insights into the effects of size and substrate confinement on morphology, composition, and crystallinity. This is important because films of different size regimes are required depending on the application [19,20]. For example, films prepared on the nanometer scale may be useful as dielectric layers in transistors [21], whereas gas filtration technologies might require micron-sized films or larger [22]. For the creation of successful MOF-based devices it should not be assumed that material properties and reactivity will remain consistent across size regimes; therefore, more comprehensive studies such as the one conducted herein are needed in order to better understand the scope and broad applicability of various host-guest interactions.

Specifically, this study compares the three different variations of HKUST-1 mentioned above: the surMOF, drop-cast thin film, and bulk powder. Metal-organic frameworks anchored to a surface are able to integrate both the versatility and tunability of MOF systems into hierarchical architectures for potential applications in electronic or sensing devices [23,24]. Bulk powders have shown promise in their capabilities for incorporation into MOF-graphene hybrid materials and real-world gas mask filtrations [15,25]. The bulk powder used in this study was synthesized, yielding large, microscale, free-standing MOF crystals [26]. The surMOF was prepared via a well-established layer-by-layer approach where nanoscale MOF crystallites were deposited in a controlled fashion onto a carboxylic acid-functionalized gold substrate [27,28]. The formation of MOF crystals on the substrate using this method was recently found to occur via a Volmer–Weber growth mechanism, giving rise to a discontinuous surface of nucleating MOF crystallites [29,30]. Lastly, the drop-cast thin film was also formed on a functionalized gold substrate, yielding a uniform film of highly-oriented crystals [26]. This is the first time that a parallel study has investigated these three MOF variations to determine whether or not they exhibit uniform behavior independent of substrate confinement and size variance.

In this study, these three variations were used to probe the interaction of HKUST-1 with ammonia gas. Metal-organic framework interactions with ammonia have been the subject of much attention over recent years because they have been show to outperform other materials in their ability to adsorb ammonia [14,31,32]. The high-performance capabilities for the HKUST-1 system have been attributed to the copper paddlewheel, a preferential binding site for the chemisorption of Lewis bases such as ammonia [33]. In addition, ammonia is also able to interact with various other parts of the MOF as well, such as the organic linkers [17]. The HKUST-1 is known to react with ammonia in different ways depending on the presence of water, making it a useful test case for examining the effect of activation and gas exposure [34]. The HKUST-1 has been shown to adsorb more ammonia under humid conditions because the ammonia can bind to the Lewis acidic Cu ions and is also able to dissolve into films of water that are contained within the pores [35]. Peterson et al. [36] showed that under dry conditions, ammonia reacted with the open-metal binding sites of the copper paddlewheel to form a copper(II)-diamine species. That same study also showed that under humid conditions, the MOF underwent a more severe degradation owing to the reaction of ammonia with the BTC linkers to form Cu(OH)_2_ and (NH_4_)_3_BTC species. After exposure under both wet and dry conditions, the ammonia was removed via heating under vacuum to regenerate the material, which could then successfully re-uptake ammonia upon subsequent exposures. The research herein examines morphology throughout multiple cycles of regeneration and re-exposure for the activated surMOF material, which adds new and practical information about the viability of using surface-constrained MOF nanomaterials. 

The interaction of ammonia with HKUST-1 has been modeled by simulations [17,37] and has been monitored via breakthrough studies [14]; IR spectroscopy [38]; X-ray crystallography [39]; nuclear magnetic resonance spectroscopy [36]; X-ray photoelectron, extended X-ray absorption fine structure, X-ray absorption near edge structure, UV−vis, electron paramagnetic resonance spectroscopies [34]; and microcalorimetry [40]. More recent work has shifted focus toward preventing or reducing MOF degradation upon ammonia exposure [41,42] and creating systems in the bulk that could function as filters in real-world applications [15]. Although positive approaches in these areas are underway, there are still fundamental aspects of the HKUST-1 ammonia interaction that need to be explored. A major shortcoming in those studies is the lack of details about the morphological changes that occur under different conditions. Therefore, for the first time ever, this study presents microscopic images for three HKUST-1 variations that show the morphological transformations upon exposure to ammonia for both the as-synthesized and activated material. This type of knowledge pertaining to changes in morphological structure has thus far been overlooked in the literature, but is essential if HKUST-1 is to be used in combination with another material, such as graphene-MOF composites [43], or integrated into device structures.

In addition, this study examines the effect of residual solvent adsorbates on the HKUST-1 ammonia interaction. For most synthetic routes towards HKUST-1, the solvent used for processing is still bound to the as-synthesized MOF either via chemisorption or physisorption [44]. It is well known that the solvent used for the processing of MOFs has an influence on the properties of the resulting framework [11]. For example, the size and porosity of HKUST-1 was shown to be altered by varying the ratio of solvents used during synthesis [45]. The presence of solvent can also hinder the ability for many MOFs to perform their desired function due to competition and blockage of binding sites [46]. The presence or absence of solvent plays a major factor in how the MOF interacts with ammonia, as evidenced by the effect of water on the reaction discussed prior. Research described herein will further explain the impact that solvent has on MOF degradation by exploring how different crystallographic and morphological changes occur depending on whether solvent molecules are within pores prior to ammonia exposure. Solvent is easily removed from HKUST-1 via activation by heat at reduced pressures, resulting in a visual color change of turquoise (as-synthesized) to dark blue (activated MOF) [10,11]. Multiple studies have confirmed that this activation process does not disrupt crystallinity [47], but potential effects on substrate morphology are not reported. Research described herein examines the morphological stability upon activation of the HKUST-1 surMOF.

Three variations of the HKUST-1 system and its interaction with ammonia gas were interrogated for both substrate-bound and free-standing materials. Ammonia gas exposure was investigated for as-synthesized and activated samples to explore the effect of residual solvent molecules. Further studies were done to gain insights into ammonia removal and re-exposure to the surMOF material. Additionally, ammonia exposure experiments, utilizing a purchased standard of HKUST-1 powder (Basolite^®^ C 300), were completed to explore the effect of additional processing solvents bound to the framework. Fourier-transform infrared spectroscopy (FT-IR), scanning electron microscopy (SEM), energy dispersive X-ray spectroscopy (EDS), scanning probe microscopy (SPM), and powder X-ray diffraction (XRD) characterized samples before and after ammonia exposure. These techniques were used to track uptake and release of ammonia, monitor changes in surface morphology, examine elemental composition, and observe effects on overall crystal structure. These fundamental studies of HKUST-1 ammonia gas interactions elucidate which effects are inherent to the MOF system, as opposed to effects that are dependent on substrate confinement or size variance, and are critical for the successful incorporation of HKUST-1 into hierarchical architectures.

## 2. Materials and Methods 

### 2.1. Materials

Copper (II) acetate monohydrate and copper (II) nitrate hemi(pentahydrate) were purchased from Fisher Scientific (Fair Lawn, NJ, USA). Absolute, anhydrous ethanol (200 proof, ACS/USP Grade) was obtained from Pharmco–Aaper (Shelbyville, KY, USA). Trimesic acid (TMA, H_3_BTC) (95%), dimethyl sulfoxide (DMSO) (spectrophotometric grade), and 16-mercaptohexadecanoic acid (MHDA) (90%) were purchased from Aldrich (St. Louis, MO, USA). The DMSO was purged with nitrogen and passed through columns of molecular sieves. Gold substrates composed of silicon wafers with 5 nm Ti adhesion layer and 100 nm Au were purchased from Platypus Technologies (New Orleans, LA, USA). Anhydrous ammonia gas was obtained from Alexander Chemical Company (Kingsbury, IN, USA).

### 2.2. Sample Preparation

Three variations of MOFs were fabricated: surface-anchored MOFs (surMOF) via layer-by-layer deposition, MOF thin films by drop-cast method, and MOF powder synthesized to produce microcrystals.

#### 2.2.1. surMOF

The surMOFs were fabricated according to literature precedent on a gold substrate functionalized by a self-assembled monolayer (SAM) using alternating, solution-phase deposition [27,28,29]. The gold substrate was first immersed in a 1 mM ethanol solution of MHDA for 1 h to form the foundational SAM that anchors the framework to the substrate. The substrate was then rinsed with ethanol and dried with nitrogen. Next, the sample was submerged in a 1 mM ethanol solution of copper acetate monohydrate. The substrate was removed after 30 min, rinsed, and dried as before. The sample was then submerged in a 0.1 mM ethanol solution of TMA for 1 h. Again, the substrate was rinsed, dried, and returned to the 1 mM copper acetate monohydrate solution. Four deposition cycles of the copper and TMA solutions yielded a film with an average thickness, roughness, and surface coverage of 10 nm, 20 nm, and 24%, respectively [29]. For all experiments herein, solutions were held at room temperature. After film formation, the substrate was characterized by SPM and IR.

#### 2.2.2. Thin Film 

The MOF thin film was prepared according to a modified drop-cast procedure [26]. The starting reagents, 2.8 mmol (0.59 g) TMA and 5.3 mmol (1.2 g) copper (II) nitrate hemi (pentahydrate), were combined in 5 mL of DMSO. This solution underwent sonication and stirring until powders were completely dissolved. A 1 mL portion of the resulting blue solution was then diluted to 5 mL by the addition of DMSO. A gold substrate that had been functionalized with a SAM of MHDA (according to the aforementioned method) was placed on a hotplate. A pipet transferred the diluted solution onto the gold to form a liquid layer that completely covered the substrate. The sample was covered with a glass beaker, the hotplate was ramped up to 100 °C over a 10-min period, and the sample was held at that temperature for an additional 10 min. As the solvent evaporated, a thin blue-green film was formed. Once the solvent was fully evaporated, the substrate was removed from the hotplate and placed onto an aluminum heat sink to cool the film to room temperature for characterization by XRD, SEM, and IR. This procedure yielded surface-confined crystals ranging in size from 1 to 10 microns. A quantitative study of crystal size and homogeneity was not undertaken.

#### 2.2.3. Powder

The MOF bulk powder was prepared according to an optimized procedure [26]. First, 2.8 mmol (0.59 g) TMA and 5.1 mmol (1.2 g) copper (II) nitrate hemi(pentahydrate) were sonicated in 5 mL DMSO solution until completely dissolved. The solution was then heated in a beaker to 100 °C on a hotplate for 2 h, which resulted in the formation of blue-green crystals. After cooling, the precipitate was sonicated in ethanol, dried by vacuum filtration, and washed with additional ethanol solvent. The powder was transferred to a test tube, suspended in dichloromethane, and centrifuged for 6 min at 3300 rpm. This process was repeated three times, after which the resulting blue powder was dried overnight under high vacuum and then stored in a vacuum desiccator to be characterized by XRD, SEM, and IR. This procedure yielded free-standing crystals ranging in size from 10 to 100 microns. A quantitative study of crystal size and homogeneity was not undertaken.

### 2.3. Ammonia Exposure 

Ammonia exposure was investigated for as-synthesized as well as activated samples. The activation process was undertaken by heating the sample under vacuum to evacuate the pores and remove any solvent (residual or coordinated). All samples that were regenerated and re-exposed underwent the same regeneration procedure.

#### 2.3.1. Activation Process

Samples were placed under high vacuum at 180 °C for 2 h. The samples were then cooled under vacuum for 30 min prior to exposure.

#### 2.3.2. Ammonia Exposure

For ammonia exposure, a sample under high vacuum at 25 °C was exposed to 360 torr of ammonia gas for 1 h. Samples exposed as-synthesized were held under high vacuum for 5 min at 25 °C prior to exposure. All samples (with and without prior activation) were exposed without breaking vacuum.

#### 2.3.3. Regeneration and Re-Exposure

To undergo regeneration, samples were held under high vacuum at 180 °C for 1 h. The samples were then cooled under vacuum for 30 min prior to characterization. Prior to re-exposure, samples were left open to the atmosphere for at least 1 h. Each sample that was re-exposed underwent the same exposure method to which it was originally exposed.

### 2.4. Standard Powder Investigation of Solvent Effects

Basolite^®^ C 300 powder was obtained from Aldrich (St. Louis, MO, USA) and was used as a standard for comparison to the HKUST-1 MOF powders fabricated as described above. The powder underwent ammonia exposures with and without activation in the manner outlined previously. Additional experimental exposures were undertaken to expose the Basolite^®^ C 300 powder to solvents prior to ammonia exposures.

#### 2.4.1. H_2_O Exposure

Standard samples underwent H_2_O exposure before subsequent ammonia exposure. The powder was exposed for 5 min to high vacuum conditions, and then for 2 h to H_2_O vapor that had been heating to 85 °C under vacuum. Next, the sample was exposed for 5 min to high vacuum. The powder was then characterized and subsequent ammonia exposure was undertaken as described in the above section “ammonia exposure”.

#### 2.4.2. DMSO Exposure

Standard samples underwent exposure to DMSO prior to subsequent ammonia exposure. A 100–250 mg portion of powder was suspended in 10–15 mL DMSO. The mixture was stirred for 2 h, after which the powder was dried via vacuum filtration and overnight house vacuum. Once dry, the powder was characterized and exposed to ammonia gas without activation.

### 2.5. Characterization

All samples underwent characterization by microscopy and infrared spectroscopy. The surMOF samples were characterized by scanning probe microscopy. The drop-cast thin films and the powder samples were characterized by scanning electron microscopy with energy dispersive spectroscopy, as well as by powder X-ray diffraction.

#### 2.5.1. Scanning Probe Microscopy (SPM) 

A Dimension Icon Scanning Probe Microscope (Bruker, Santa Barbara, CA, USA) that operated in peak force tapping mode was used to obtain several images (512 × 512 pixels) for each sample, both before and after exposures, at 5 μm × 5 μm and 500 nm × 500 nm. Etched silicon tips, SCANASYST-AIR (Bruker, Santa Barbara, CA, USA), with a spring constant range of 0.2–0.8 N/m and a resonant frequency range of 45–95 kHz were used. Scan parameters were as follows: 1 Hz scan rate, 12 μm z-range, 250–370 mV amplitude set point, and 100–450 mV drive amplitude. Image analysis was carried out by Nanoscope Analysis software (Bruker, Santa Barbara, CA, USA).

#### 2.5.2. Infrared Spectroscopy (IR)

Infrared spectra were collected from 3800–600 cm^−1^ in ATR (Attenuated Total Reflectance) mode and used a Thermo Scientific Nicolet iS50 instrument. The spectra were collected at a resolution of 4 cm^−1^ and used a bare gold substrate as the background for the surMOF studies and ambient air as the background for the powder and thin-film studies.

#### 2.5.3. Scanning Electron Microscopy (SEM) and Energy Dispersive X-ray Spectroscopy (EDS)

The SEM images were collected by a Hitachi TM-3000 tabletop microscope and used an accelerating voltage of 15 kV with the detection of back-scattered electrons. The EDS data were collected at this voltage by this microscope coupled with a Bruker XFlash MIN SVE detector and scan generator for EDS capability.

#### 2.5.4. Powder X-ray Diffraction (XRD)

The XRD patterns were collected by a Rigaku Miniflex X-ray diffractometer and used Cu Kα radiation at 30 kV and 15 mA. Data were collected from 10.00° to 79.99° for the powder and 10.00° to 34.00° for the drop-cast thin film (range selected to avoid the intense gold peaks from the underlying substrate). All samples were analyzed at a sampling width of 0.03° and scan speed of 3°/min. 

## 3. Results and Discussion

To investigate how ammonia interacts with the HKUST-1 metal-organic framework, three different variations of the material (surMOF, drop-cast thin film, and powder) were exposed to ammonia; and the effect was monitored via microscopy, IR, and powder XRD. For these MOF variations, both as-synthesized and activated (heated under vacuum to remove species within pores) samples were investigated. The SPM characterization was undertaken to monitor how ammonia interacts with the surMOF deposited by a layer-by-layer protocol onto a gold surface functionalized with a self-assembled monolayer. The SEM characterization was used to monitor both a thin-film framework fabricated via drop-casting onto a functionalized gold surface as well as the synthesized bulk powder of microcrystals. Infrared spectroscopy was utilized in all three cases to track the uptake and release of ammonia. For the thin film and powder, powder XRD patterns were collected to gain further insights into how ammonia affects the overall crystal structure; and EDS was used to examine elemental composition. Beyond monitoring the effect of the initial ammonia exposure, both SPM and IR were used to understand what happens when ammonia is removed and re-introduced to the surMOF material. To further investigate the findings from the aforementioned experiments and explore the effect of different processing solvents, experiments utilizing a purchased standard of HKUST-1 powder (Basolite^®^ C 300) were conducted.

### 3.1. Morphological Characterization

**Figure 2:**
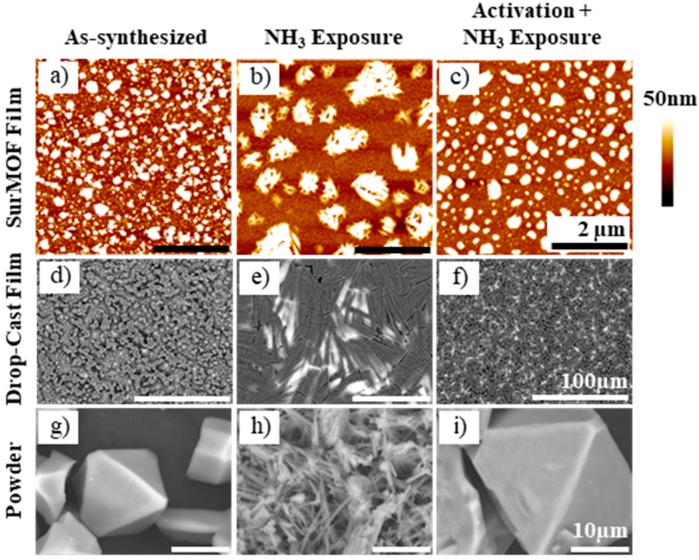
Top: Representative SPM images of HKUST-1 surMOFs (**a**) before exposure to NH_3_; (**b**) after exposure without activation; and (**c**) after exposure with prior activation. Middle: SEM images of HKUST-1 drop-cast thin film (**d**) before exposure to NH_3_; (**e**) after exposure without activation; and (**f**) after exposure with prior activation. Note: Due to high conductivity, underlying gold substrate appears bright in SEM images (especially prevalent in (**e**) and (**f**)). Bottom: SEM images of HKUST-1 powder (**g**) before exposure to NH_3_; (**h**) after exposure without activation; and (**i**) after exposure with prior activation. All scale bars in (**a**–**c**) are 2 µm, in (**d**–**f**) are 100 µm, and in (**g**–**i**) are 10 µm.

Integral to understanding how the material is affected by its interaction with ammonia, SPM and SEM characterization reveal the morphological structure of the surMOF, thin film, and powder HKUST-1 variations. Images of these structures before ammonia exposure, after exposure without activation, and after exposure with prior activation are shown in Figure 2. In all three cases, when the framework was exposed to ammonia without activation, the material undergoes a dramatic morphological change. The surface of the surMOF material changed from smaller, evenly distributed crystallites (Figure 2a) to larger bundles of nanowire-like structures (Figure 2b). A similar change of surface morphology was observed in the thin film (Figure 2d,e). The powder material also underwent a complete morphological change from octahedral shaped crystals (Figure 2g) to the nanowire-like structures (Figure 2h).

A minor morphological change was observed when the framework was activated prior to ammonia exposure. The surMOF underwent a slight morphological change where the sharpness of the nanocrystallite features were reduced; and the size of the isolated structures on the surface increased (Figure 2c) relative to the film features before exposure (Figure 2a). The thin film and the powder materials did not undergo significant morphological changes after exposure to ammonia with activation (Figure 2f,i).

These images and observations provide new and important information as to how ammonia affects the morphology of the HKUST-1 crystals dependent on whether or not the material was activated (removing water or residual solvent) before the framework was exposed to the gas. When the sample was not activated, the ammonia-framework interaction resulted in a dramatic morphological change that was not observed when the framework was activated prior to exposure. This result is consistent with the findings of Peterson et al. [36] that the presence of water or residual solvent alters the reaction of the MOF with ammonia. Based on the morphological changes observed and the conclusions by Peterson, it can be hypothesized that the nanowires formed by exposure without prior activation could be Cu(OH)_2_ crystallites and that the changes (or lack thereof) observed in the samples exposed with prior activation could be due to the formation of a copper(II)-diamine species. Regardless of the products formed, the images clearly show that different interactions are taking place dependent on whether the sample was activated.

Further, it was confirmed that the heating process involved did not cause a significant morphological change on the surMOF, which is an important observation because MOF materials are often activated prior to being used. This was shown by an experiment in which the as-synthesized sample was characterized following the activation process without subsequent ammonia exposure (shown in Section 3.5).

### 3.2. Compositional Characterization

**Figure 3:**
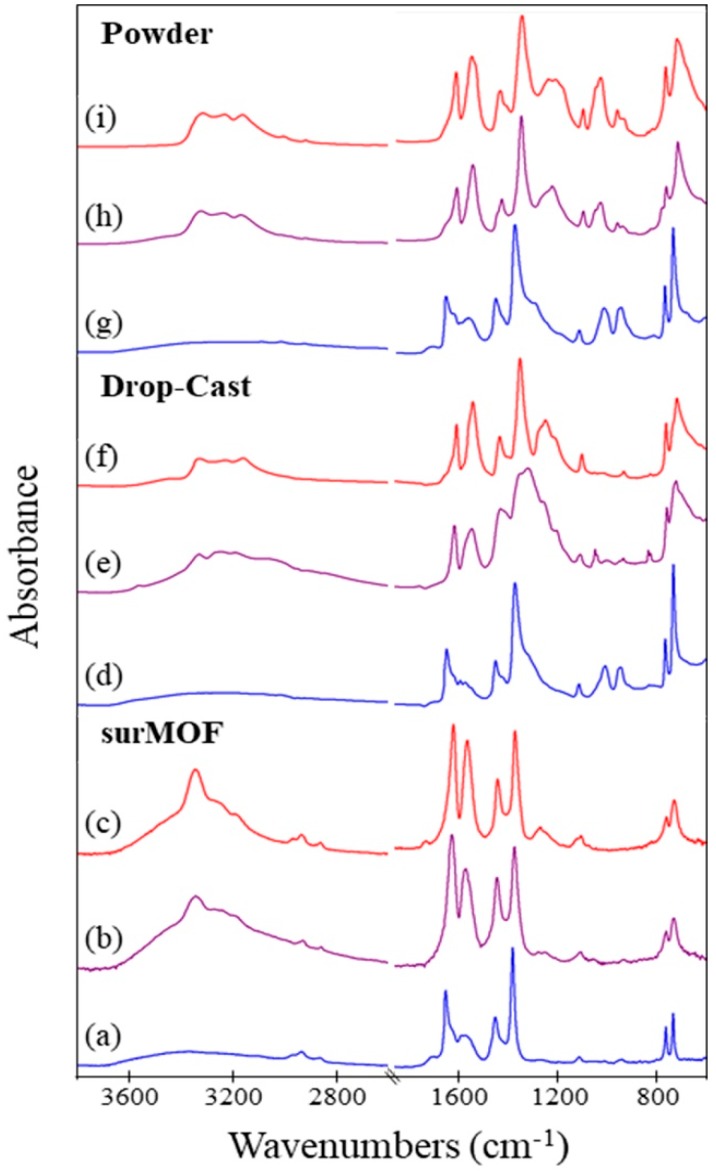
Infrared (IR) spectra of HKUST-1: (**a**) surMOF before exposure to NH_3_; (**b**) surMOF after exposure without activation; (**c**) surMOF after exposure with prior activation; (**d**) drop-cast thin film before exposure to NH_3_; (**e**) drop-cast thin film after exposure without activation; (**f**) drop-cast thin film after exposure with prior activation; (**g**) powder before exposure to NH_3_; (**h**) powder after exposure without activation; and (**i**) powder after exposure with prior activation.

Infrared spectra were obtained to investigate the chemical composition of the framework. This characterization technique was utilized to monitor uptake of ammonia and to examine how this exposure affected the framework. A comparison was conducted for when the sample underwent exposure to ammonia both without and with activation. Spectra of the framework before ammonia exposure, after exposure of an as-synthesized sample, and after exposure of an activated sample are shown in Figure 3 for the surMOF, thin film, and powder variations of the framework. The EDS was obtained for the powder and thin film samples to investigate the chemical identity of compounds captured within the framework, such as the DMSO solvent or the ammonia gas.

Characteristic IR peaks for HKUST-1 are present in all samples prior to exposure [27,34,38]; and a few small differences between the MOF variations are observed (Figure 3a,d,g). The asymmetric COO^-^ stretch (~1650 cm^−1^) and the C–C aromatic vibration peak, corresponding to the linker molecule (~1440 cm^−1^), are present in all spectra. All spectra for the as-synthesized materials also contain a very broad peak at ~3000–3400 cm^−1^, indicative of water or deposition solvent within the framework (Figure 3a,d,g). Peaks at 2930 cm^−1^ and 2855 cm^−1^ are only present in the surMOF sample and correspond to the C–H stretch of the underlying self-assembled monolayer on the gold substrate (Figure 3a–c) [48]. Peaks at 950 cm^−1^ and 1000 cm^−1^ are present only in the thin film and powder spectra (Figure 3d,g) and correspond to the DMSO solvent, indicating that it is present within the framework. The DMSO also has broad peaks around ~3000 cm^−1^, which occur at the same vibrational frequency as water [49]. For both the as-synthesized thin film and powder sample, EDS data confirm the presence of the DMSO deposition solvent, showing a 1:1 ratio of the Cu:S (SI Figures 1 and 4). This suggests that each open axial copper paddlewheel position may be occupied by one DMSO ligand.

All of the data gleaned from the IR spectra indicate that ammonia was present after exposure regardless of prior activation and HKUST-1 variation. Changes occurred in the spectra when the framework was exposed to ammonia (Figure 3b,c,e,f,h,i) consistent with literature observations [34]. A new distinct peak appeared around 3300 cm^−1^ that corresponds to the N-H stretch of ammonia on the framework [50]. This was true regardless of whether the framework was activated prior to exposure, which indicated that the uptake of ammonia into the framework was not dependent on activating the framework. Other changes that occurred upon exposure to ammonia were the shifting of the peak at 1650 cm^−1^ to 1625 cm^−1^, the increase in intensity of peak at 1560 cm^−1^, the appearance of two peaks in the 1260–1210 cm^−1^ range, and the broadening/shifting of the 1370 cm^−1^ and 1450 cm^−1^ peaks. All of these observances are in accord with previous studies investigating the effect of ammonia gas on the HKUST-1 framework [34]. After ammonia exposure for both as-synthesized and activated samples, EDS for the thin film and powder variations confirmed the presence of nitrogen and the removal of sulfur (SI Figures 2, 3, 5 and 6). (Note that the low-energy -ray peak associated with nitrogen in the crowded region with carbon and oxygen did not permit quantitative analysis of the nitrogen composition, but did qualitatively confirm its presence.) The data show that the ammonia gas displaced any solvent molecules present within the as-synthesized framework.

### 3.3. Crystal Structure Characterization

Powder XRD data were obtained for the thin film and powder systems, but could not be obtained for the surMOF films investigated in this study due to the low density of material bound to the surface. The similarities observed via microscopy and IR analysis for all three variations suggest that crystal structure findings for the thin film and powder likely translate to the surMOF variation.

**Figure 4:**
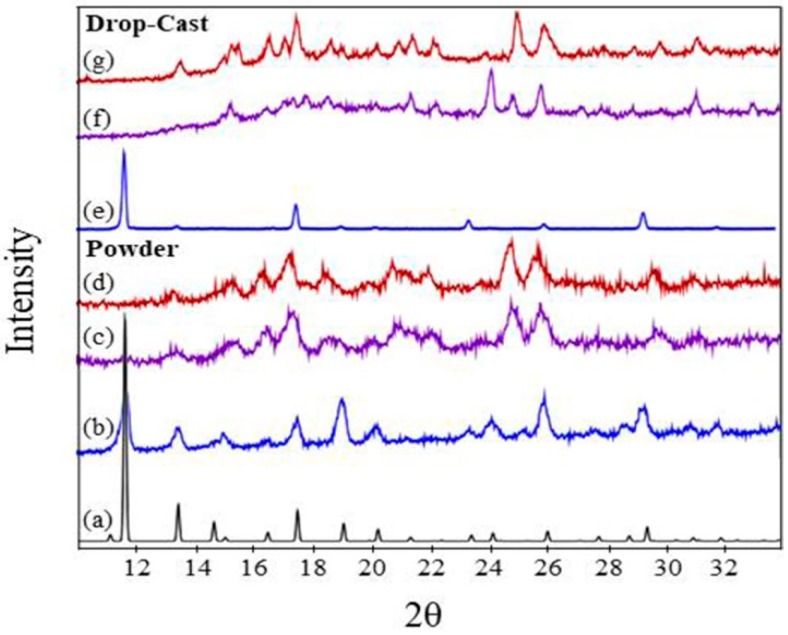
XRD patterns of (**a**) reference for HKUST-1 powder (ICDD pdf number 00-062-1183); (**b**) powder before exposure to NH_3_; (**c**) powder after exposure without activation; (**d**) powder after exposure with prior activation; (**e**) drop-cast thin film before exposure to NH_3_; (**f**) drop-cast thin film after exposure without activation; and (**g**) drop-cast thin film after exposure with prior activation.

Powder XRD was important for confirming the HKUST-1 structure for the synthesized thin film and bulk samples, as well as for characterization of the crystal structure after ammonia exposure. Patterns for the material before ammonia exposure, after exposure without activation, and after exposure with prior activation are shown in Figure 4 for the thin film and powder variations of the framework. The peaks corresponding to the thin film and powder material pre-exposure (Figure 4b,e) match well with the reference pattern (Figure 4a), indicating that the HKUST-1 crystal structure was obtained. The MOF deposited as a thin film (Figure 4e) demonstrates a preferred (111) crystal orientation. Noteworthy is that the XRD for the powder has broader peaks in comparison to the film, revealing the highly crystalline nature of the film. 

Ammonia exposure with and without activation resulted in a change in crystal structure as observed by powder XRD (Figure 4c,d,f,g); and the resulting patterns are in general agreement with previous studies investigating the effect of ammonia on HKUST-1 [10,36,46]. For the HKUST-1 powder, the patterns for both as-synthesized and activated samples after ammonia exposures are consistent with one another (Figure 4c,d). This indicates that the activation step did not prevent the change in the crystal structure, despite what the SEM revealed with the crystal morphology remaining consistent. The same was observed for the as-synthesized and activated HKUST-1 thin films (Figure 4f,g). These observations highlight the importance of monitoring changes in both the morphology and the crystal structure, demonstrating that changes in crystallographic structure does not necessitate significant changes in substrate morphology. When comparing the ammonia exposures for the film and powder, the broadness of the peaks for the ammonia exposures to the film and powder are different, but the locations are consistent, revealing that the same crystal structure transformation is observed. The differences in the patterns are that the powder sample has broad peaks, while the film sample has sharper and therefore more distinguishable peaks. This is consistent with the samples before exposure with broader peaks observed for the synthesized powder (Figure 4b) and sharper peaks observed for the synthesized film (Figure 4e).

### 3.4. Standard Powder Investigation of Solvent Effects

Experiments with a standard HKUST-1 powder were undertaken in order to investigate a sample with and without residual solvent ligands within the framework. For the synthesized thin film and powder HKUST-1 samples, DMSO was found to be present in the as-synthesized materials and is likely coordinated to the open copper paddlewheel site. For the surMOF HKUST-1 sample, water is within the framework and likely bound at this same site. By conducting the experiments with a standard powder obtained commercially, this study investigated (1) the outcome of ammonia exposures on this standard powder void of residual solvent; (2) the effect of residual water and DMSO on this powder; and (3) the outcome of an ammonia exposure after the water or DMSO solvent were present within the framework.

#### 3.4.1. Standard Powder as Received

**Figure 5:**
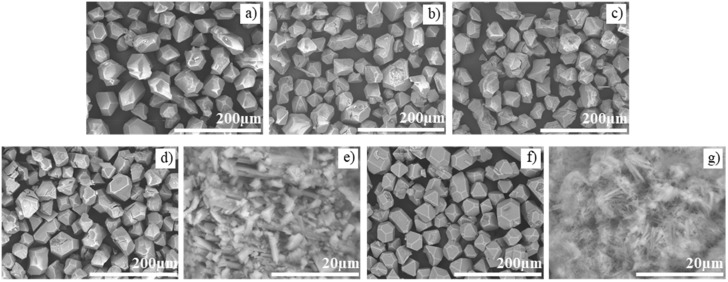
Representative SEM images of HKUST-1 powder (obtained commercially as Basolite^®^ C 300) (**a**) before gas exposure; (**b**) after NH_3_ exposure without activation; (**c**) after NH_3_ exposure with prior activation; (**d**) after H_2_O vapor exposure; (**e**) after H_2_O vapor and subsequent NH_3_ exposure without activation; (**f**) after DMSO exposure; (**g**) after DMSO and subsequent NH_3_ exposure without activation.

**Figure 6:**
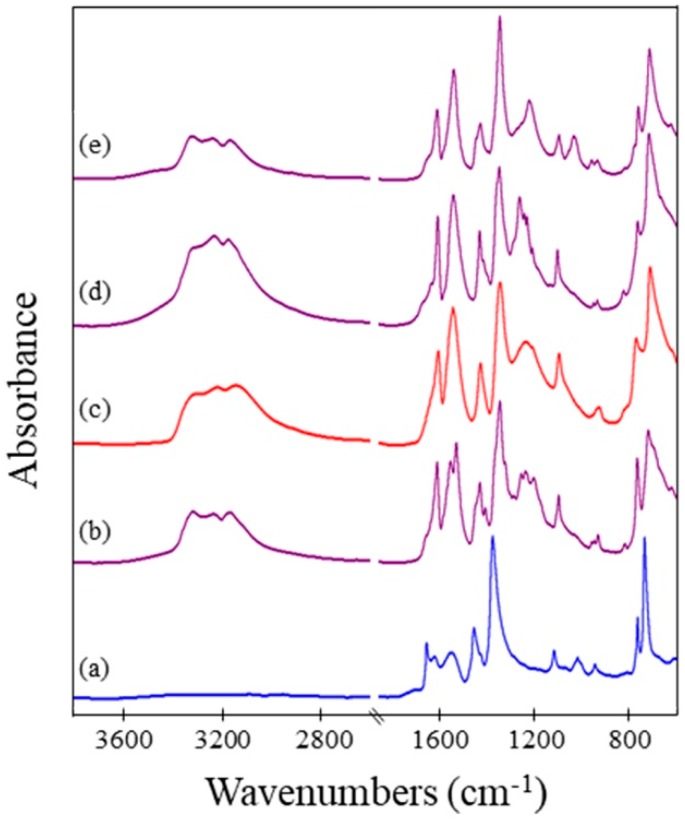
IR spectra of HKUST-1 powder (obtained commercially as Basolite^®^ C 300) (**a**) before gas exposure; (**b**) after NH_3_ exposure without activation; (**c**) after NH_3_ exposure with prior activation; (**d**) after H_2_O vapor and subsequent NH_3_ exposure without activation; (**e**) after DMSO and subsequent NH_3_ exposure without activation.

**Figure 7:**
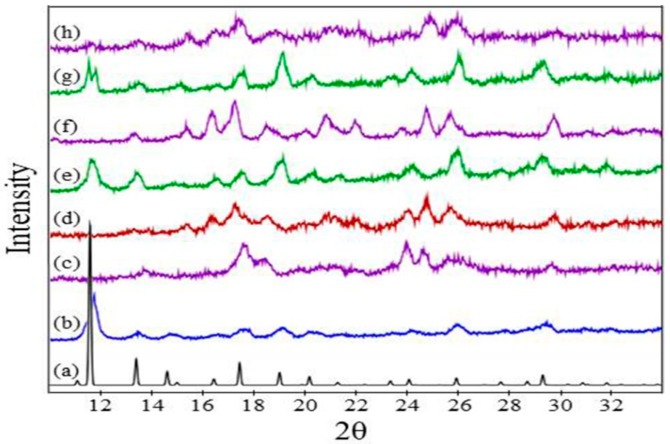
XRD patterns of (**a**) reference pattern for HKUST-1 powder (ICDD pdf number 00-062-1183); (**b**) commercially obtained HKUST-1 powder (Basolite^®^ C 300) before gas exposure; (**c**) after NH_3_ exposure without activation; (**d**) after NH_3_ exposure with prior activation; (**e**) after H_2_O vapor exposure; (**f**) after H_2_O vapor and subsequent NH_3_ exposure without activation; (**g**) after DMSO exposure; (**h**) after DMSO and subsequent NH_3_ exposure without activation.

The obtained standard powder was found to be consistent with literature precedent for HKUST-1 by SEM [11], IR [34], and XRD [10,36,46] characterization (Figure 5a, Figure 6a and Figure 7b). The SEM analysis of the crystal morphology for the standard powder was consistent with the powder synthesized by the procedure described herein. The EDS data showed no evidence of nitrogen present and minimal (2%–3%) sulfur (SI Figure 7). It is noteworthy that the standard powder matched the peak intensities of the HKUST-1 reference pattern (Figure 7a,b) more so than the synthesized powder shown in Figure 4b. 

Exposures of ammonia gas for as-synthesized and activated samples were both successful, as IR confirmed the presence of ammonia (Figure 6b,c); and EDS data qualitatively showed similar uptake of nitrogen for both ammonia exposures (SI Figures 8 and 9). The SEM images reveal no morphological changes after either type of exposure (Figure 5b,c), suggesting that the standard powder was activated as received. (Note that the presence of silicon was observed via EDS (SI Figure 7), and this diatomaceous earth was observed in SEM images, which was likely an active desiccant to help maintain activation.) Despite the lack of morphological changes, the crystal structure observed by XRD did change upon exposure to ammonia (Figure 7b–d). The XRD patterns for the standard powder without and with activation (Figure 7c,d) are consistent with one another as well as with the XRD patterns for the ammonia exposures of the as-synthesized HKUST-1 powder (Figure 4c,d).

#### 3.4.2. Standard Powder with Water Exposure

After exposing the standard powder to water vapor, the crystal morphology observed via SEM was unchanged (Figure 5d), and the crystal structure was maintained according to XRD (Figure 7e). Noteworthy, the peak intensities observed in the diffraction pattern were more similar to the synthesized powder (Figure 4b) rather than the standard powder as-received (Figure 5b). This is indicative that the water is bound to the framework of the standard powder after water exposure.

For this standard powder exposed to water vapor, an ammonia exposure resulted in a change in crystal morphology observed by SEM (Figure 5e), the uptake of ammonia detected by EDS (SI Figure 10) and IR (Figure 6d), and a change in crystal structure seen by XRD (Figure 7f). The morphology for this sample that had undergone an ammonia exposure after water vapor exposure (Figure 5e) was similar to that of the as-synthesized HKUST-1 powder upon exposure to ammonia (Figure 2h). The XRD data after ammonia exposure for this sample (Figure 7f) was consistent with the exposures on the standard powder (Figure 7c,d) and the synthesized powder (Figure 4c,d).

#### 3.4.3. Standard Powder with DMSO Exposure

After exposing the standard powder to DMSO, the crystal morphology was unchanged as observed via SEM (Figure 5f). The crystal structure was maintained with the same small change in peak intensity (Figure 7g) as observed for the standard powder after water exposure (Figure 7e), which again was similar to that of the synthesized powder (Figure 4b). The IR shows peaks at approximately 950 cm^−1^ and 1000 cm^−1^ which are consistent with DMSO. The EDS shows a 1:1 ratio of Cu:S (SI Figure 8), which correlates with the same ratio found for the synthesized powder. This IR and EDS data suggests 1 DMSO may be coordinated to each axial copper paddlewheel site.

Upon subsequent ammonia exposure, IR and EDS data indicated that ammonia was captured (Figure 6e) (SI Figures 11 and 12). The SEM showed that the crystal morphology changed (Figure 5g) in a manner consistent with what had been observed for all ammonia exposures of as-synthesized samples (Figure 2b,e,h). The resulting XRD pattern (Figure 7h) is consistent with the patterns for other ammonia exposures (Figure 7c,d,f and Figure 4c,d), especially those of the synthesized powder (Figure 4c,d). For the standard powder with water exposure before ammonia exposure, the XRD peaks (Figure 7f) were sharper, correlating with the size of the crystals observed by SEM (Figure 5f) relative to those for both the standard powder exposed to DMSO and the synthesized powder after ammonia exposure (Figure 5g and Figure 2h, respectively).

#### 3.4.4. Discussion of Solvent Effects on Standard Powder 

This study shows that the presence of different solvent ligands results in the loss of crystal morphology after ammonia exposure, while the crystal structure is essentially the same independent of the solvent molecules presence. Why does the presence of the ligand result in loss of the crystal morphology? Based on this research, it is hypothesized that this may be that the ligand is bound to the copper paddlewheel, destabilizing the structure. This is specifically supported by the change in the XRD pattern of the standard powder after it is exposed to water and DMSO. The change in peak intensities may be indicative of this destabilization. In addition, if water or solvent is in fact bound to the copper paddlewheel as hypothesized, then this creates a competition for binding when ammonia is introduced [37]. This competition likely changes the way ammonia is able to interact with the various parts of the framework, explaining why the morphology change is different depending on the absence or presence of solvent. By activating the HKUST-1 before ammonia exposure, one is not able to prevent the change in crystal structure but is able to prevent the dramatic change in morphology, which remained unknown until now. This is advantageous because morphological changes destroy film continuity and would affect the packing and interaction of the powder within a composite material or device architecture. Understanding and preventing this degradation is fundamental toward the integration of MOF materials into hierarchical structures for the realization of their potential in applications as thin film sensors or for industrial gas sequestration.

### 3.5. Characterization of Regeneration and Re-exposure

This research has shown that the presence of residual solvent disrupts the morphology of three HKUST-1 variations upon exposure to ammonia and that this disruption can be prevented by activation prior to initial gas exposure. Will the original morphology be preserved upon the removal of the ammonia from within the framework and, if so, then what about after a second round of gas exposure? Toward future applications, it is necessary for the material to be morphologically stable not just upon initial exposure, but also for the ammonia uptake to be reversible for multiple cycles of exposure, removal, and re-exposure. This investigation was undertaken for the surMOF HKUST-1 variation with SPM and IR. This was done to determine if the ammonia can be removed after exposure and then whether the framework can re-uptake ammonia upon a subsequent exposure.

**Figure 8:**
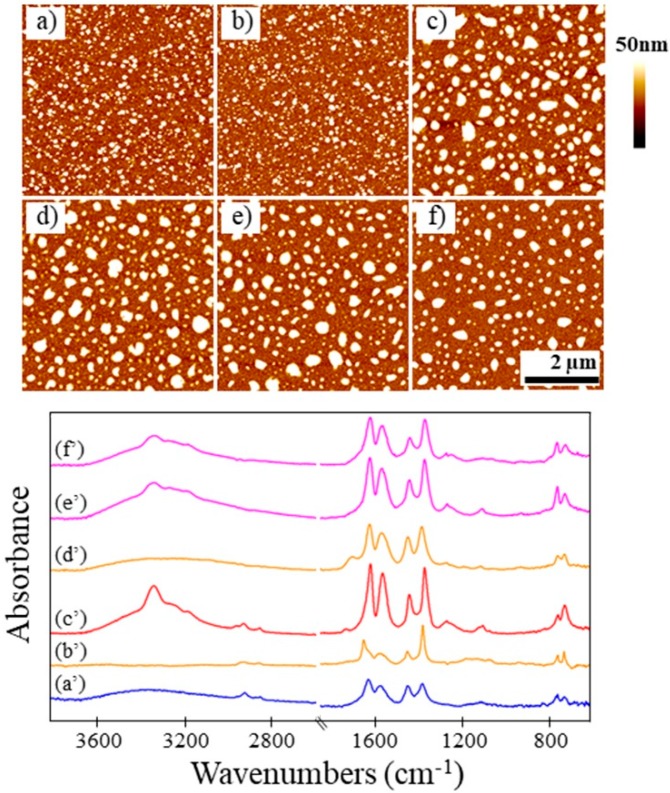
Top: Representative SPM images of HKUST-1 surMOFs (**a**) before exposure to NH_3_; (**b**) after activation; (**c**) after exposure with prior activation; (**d**) after regeneration; (**e**) after second exposure with activation; and (**f**) after sixth exposure with activation. Bottom: Corresponding IR spectra (**a’**–**f’**).

Scanning probe microscopy images and IR spectra in Figure 5 show that the activated surMOF (Figure 8b) remained morphologically stable after it was exposed to ammonia once (Figure 8c), regenerated by the removal of ammonia (Figure 8d), and exposed to ammonia a second and sixth time (Figure 8e,f). The SPM images reveal a minor loss in the sharpness of the features after the initial exposure, but the morphology did not change again even after multiple rounds of regeneration and re-exposure. The IR data support that the ammonia was removed by the regeneration process and re-incorporated upon re-exposure. The peak in the IR spectra (~3300 cm^−1^) corresponding to ammonia was no longer present after regeneration (Figure 8d’), and the peak was again observed after the second (Figure 8e’) and sixth (Figure 8f’) exposures, indicating successful removal and re-addition of ammonia onto the framework. The observation that ammonia can re-bind to the substrate even after the material undergoes a morphological change is consistent with the results of Peterson et al., which showed that ammonia could still interact with the substrate after undergoing its initial changes in structure and composition [36].

## 4. Conclusions

For the successful incorporation of HKUST-1 into hierarchical architectures, fundamental research is necessary to investigate the effect of heat and gas on the material structure. This study explored how the morphology and crystal structure of the MOF respond to ammonia gas exposure, yielding insights into the effect of different residual solvent molecules within the HKUST-1 framework. In studying three variations of HKUST-1 (surMOF, drop-cast thin film, bulk powder), it was found that all responded similarly, independent of substrate confinement and size variance. While microscopy demonstrated a dramatic change in the morphological structure upon ammonia exposure for the as-synthesized material, activation of the framework by prior heating was demonstrated to rid the framework of solvent molecules and thus mitigates the disruption. An alteration in crystal structure, observed by XRD, was shown to occur in both the drop-cast film and powder, regardless of activation. Additionally, IR supported the successful uptake of ammonia, independent of activation prior to exposure. For activated surMOF films, ammonia uptake was shown to be reversible, permitting removal and recycling upon additional exposures, preserving framework morphology throughout. These findings provide new information about the HKUST-1 ammonia interaction specific to morphological stability and crystal structure change.

Building upon the research herein, additional avenues of study will be explored to expand the versatility and performance capability of HKUST-1 and other MOF systems for gas capture. Morphological stability studies involving exposure with additional gas varieties, such as arsine or hydrogen sulfide, are of interest to further understand the morphological impact induced by the host-guest interaction within both free-standing and surface-anchored HKUST-1 variations. Literature precedent has shown by XRD that HKUST-1 degrades upon arsine exposure without prior activation, whereas hydrogen sulfide is observed to disrupt the framework with or without prior activation [15]. Additional MOF systems, such as MOF-5, MOF-177, and UiO-66, shall be observed to determine morphological stability upon ammonia exposure with and without activation. Prior studies demonstrated that UiO-66 was stable according to XRD upon activated exposure [51], while both MOF-5 and MOF-177 systems were observed to degrade via XRD [52]. This area of research will yield further understanding regarding the potential capabilities and limitations of MOF materials, continuing toward the goal of incorporating MOF assemblies as smart interfaces for sensing applications or selective gas adsorption devices.

## Supplementary Materials

The following are available online at http://www.mdpi.com/2079-4991/8/9/650/s1, SI Figures 1–12: Energy Dispersive X-ray Spectroscopy Data.
