# Supplementary material for: Comparison of Surface-Bound and Free-Standing Variations of HKUST-1 MOFs: Effect of Activation and Ammonia Exposure on Morphology, Crystallinity, and Composition
Source: Nanomaterials (Basel). 2018 Aug 23;8(9):650. doi: 10.3390/nano8090650 (PMC6164254; doi:10.3390/nano8090650)
Supplement: Supplementary file 1 [file nanomaterials-08-00650-s001.pdf]

Supporting Information: "Comparison of Surface-Bound and Free-Standing Variations of HKUST-1 MOFs: Effect of Activation and Ammonia Exposure on Morphology, Crystallinity, and Composition"

by Brandon H. Bowser, Landon J. Brower, Monica L. Ohnsorg, Lauren K. Gentry, Christopher K. Beaudoin, Mary E. Anderson*

Energy Dispersive X-ray Spectroscopy Characterization

Energy dispersive x-ray spectroscopy (EDS) was utilized alongside scanning electron microscopy (SEM) to characterize bulk powder and drop-cast film samples of HKUST-1. While SEM elucidated the morphological properties of the material, EDS yielded qualitative and quantitative elemental composition information. The coordination of the DMSO solvent within the HKUST-1 framework in a 1:1 ratio with the copper ions was a significant EDS finding for the as-synthesized powder and drop-cast film. Upon exposure to ammonia gas, the reduction of S (relative to the Cu) within the framework is quantitatively measured. The uptake of ammonia can be observed by the appearance/increase of a N peak within the EDS spectra. Due to the overlap of the N peak with that of C and O (also found within the framework), the amount of nitrogen within the framework could not be quantitatively determined. The $K_{\alpha1}$ energy levels for C, N, O, Si, S, and Cu are all given in SI Table 1. The $K_{\alpha1}$ was what was used to quantitatively determine elemental compositions (i.e. % Cu and %S).

SI Table 1. X-Ray Wavelengths in keV

Element	Energy (keV)
C	0.277
N	0.392
O	0.525
Si	1.740
S	2.309
Cu	8.046

As-synthesized HKUST-1 Powder

Shown in SI Figure 1a is the full spectrum collected for the as-synthesized HKUST-1 powder with the Cu and S peaks identified. SI Figure 2a and SI Figure 3 provide spectra for powder after exposure to ammonia without and with prior activation, respectively. Noteworthy is that the size of the S peak relative to Cu decreases significantly. Quantitatively, the decrease in the amount of S is ~90%.

SI Figure 1b and 2b highlight the 0-3 keV region of the spectra and identify the C, N, O, S, and Cu x-ray lines. The increase in the N peak can be readily observed at this resolution. Note that the increase in peak intensity in this region (~0.4 keV) can also be observed in SI Figure 2a

and 3 in comparison to SI Figure 1a. This qualitative increase in the N peak is indicative of the ammonia uptake that was confirmed by IR analysis (Figure 3).

As-synthesized HKUST-1 Drop-Cast Thin Film

SI Figure 4 provides the full spectrum collected for the as-synthesized HKUST-1 drop-cast film, and SI Figure 5 and 6 display spectra for the drop-cast film after ammonia exposures without and with prior activation, respectively. Note that the Cu and S percentages are quantitatively determined and given within the caption, while the nitrogen peak at ~0.4 eV can be observed for qualitative comparison. The decrease in S and increase in N are confirmation that the paddlewheel adsorbates are being displaced by the uptake of ammonia. This is also confirmed by IR analysis (Figure 3).

Standard HKUST-1 Powder to Examine Solvent Effects

SI Figure 7a displays the full spectrum collected for the HKUST-1 standard powder (Basolite C), and SI Figure 7b highlights the 0-3 keV region and permits the identification of C, N, O, Si, S, and Cu. Very minor amounts of N, Si, and S are observed. SI Figure 8 and 9 exhibit spectra for this standard powder after ammonia gas exposure with and without activation, respectively. Note the 0-3 keV region is shown so that the qualitative increase in N is clearly apparent. No quantitative analysis is undertaken for these spectra.

SI Figure 10 corresponds to the HKUST-1 standard powder that had undergone the introduction of water vapor prior to ammonia activation. This spectrum is also shown highlighting the 0-3 keV region for ease of identifying the uptake of ammonia via the increase in the N peak. No quantitative analysis is undertaken for this spectrum.

SI Figure 11 provides the full spectrum for the HKUST-1 standard powder that had undergone the introduction of DMSO solvent. The amount of Cu and S are quantitatively determined, and this spectrum should be compared to SI Figure 1, as the amount of S relative to Cu for the as-synthesized HKUST-1 powder is similar. SI Figure 12 represents this sample after ammonia exposure. The decrease in S is quantitatively determined, and the increase in N can be qualitatively observed by comparing the spectral peaks at ~0.4 keV.

a)

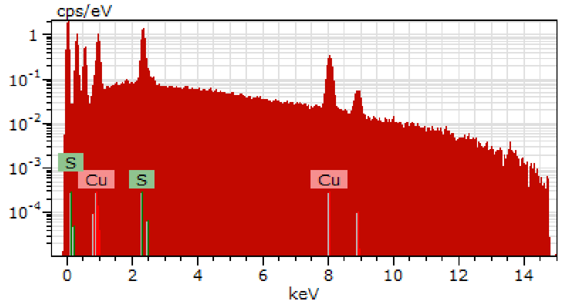

b)

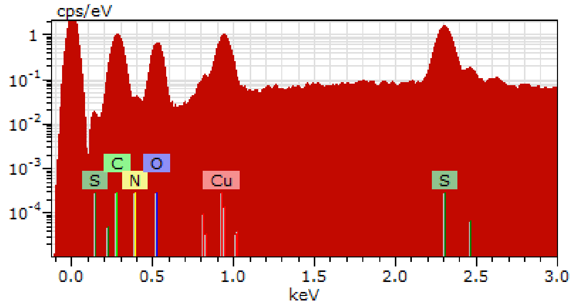

SI Figure 1. As-synthesized HKUST-1 powder. Quantification yielded 54% Cu and 46% S at 8.0 keV and 2.3 keV, respectively.

a)

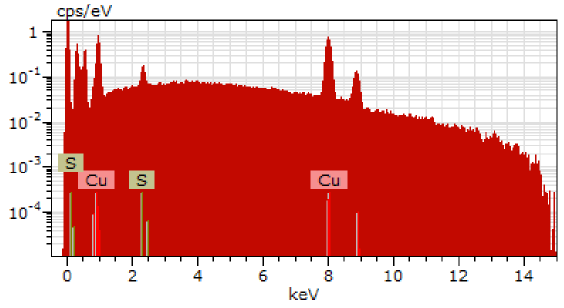

b)

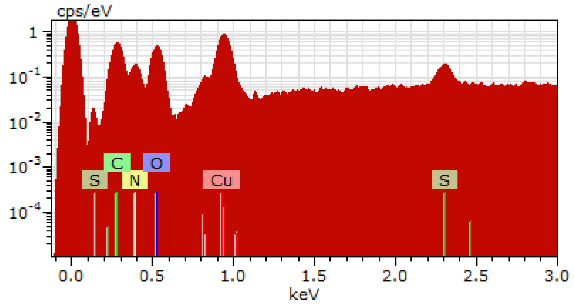

SI Figure 2. As-synthesized HKUST-1 powder after exposure to ammonia. Note that in comparison to SI Figure 1, the S peak at 2.3 keV is significantly reduced and N peak at ~0.4 keV becomes apparent. (94% Cu, 6% S)

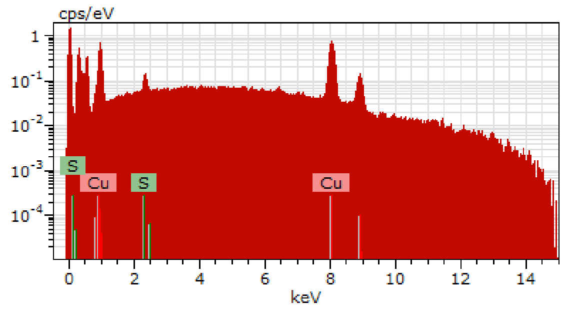

SI Figure 3. As-synthesized HKUST-1 powder after activation and subsequent exposure to ammonia. Note that in comparison to SI Figure 1, the S peak at 2.3 keV is significantly reduced, and N peak at ~0.4 keV becomes apparent. (95% Cu, 5% S)

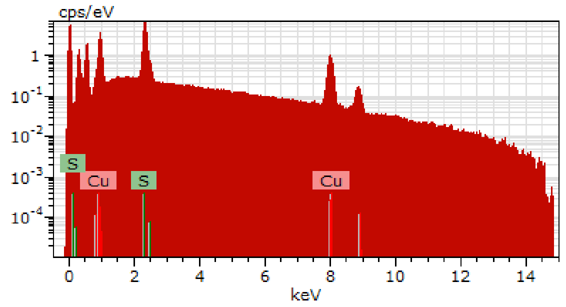

SI Figure 4. As-synthesized HKUST-1 drop-cast thin film. Quantification yielded 48% Cu and 52% S.

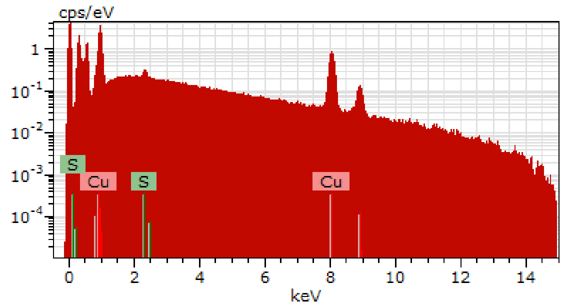

SI Figure 5. As-synthesized HKUST-1 drop-cast thin film after exposure to ammonia. Note that in comparison to SI Figure 4, the S peak at 2.3 keV is significantly reduced and N peak at ~0.4 keV becomes apparent. (98% Cu, 2% S)

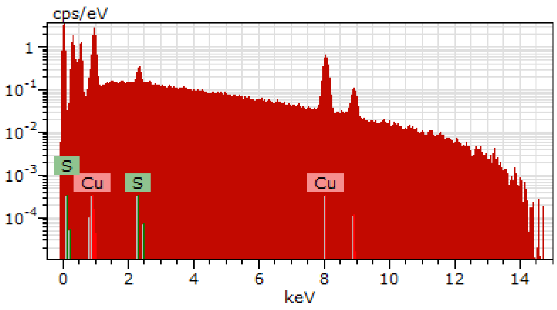

SI Figure 6. As-synthesized HKUST-1 drop-cast thin film after activation and subsequent exposure to ammonia. Note that in comparison to SI Figure 4, the S peak at 2.3 keV is significantly reduced and N peak at ~0.4 keV becomes apparent. (95% Cu, 5% S)

a)

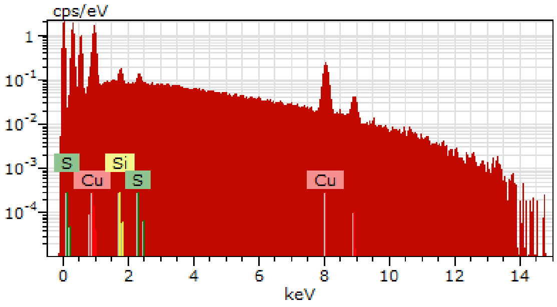

b)

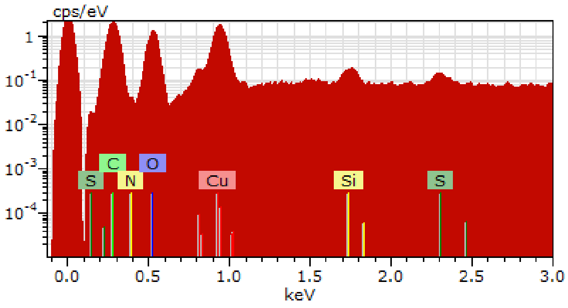

SI Figure 7. Standard HKUST-1 powder as-received. Quantification yielded 92% Cu and 3% S. (Note 5% Si was observed due to the presence of diatomaceous earth.)

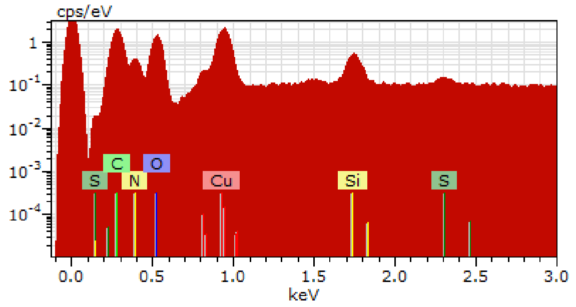

SI Figure 8. Standard HKUST-1 powder after activation and subsequent exposure to ammonia. Note the increase in the N peak at ~0.4 keV relative to SI Figure 7b.

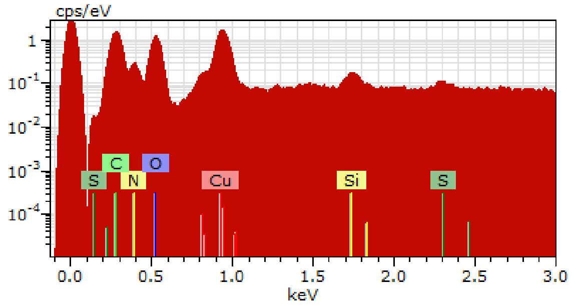

SI Figure 9. Standard HKUST-1 powder after exposure to ammonia. Note the increase in the N peak at ~0.4 keV relative to SI Figure 7b.

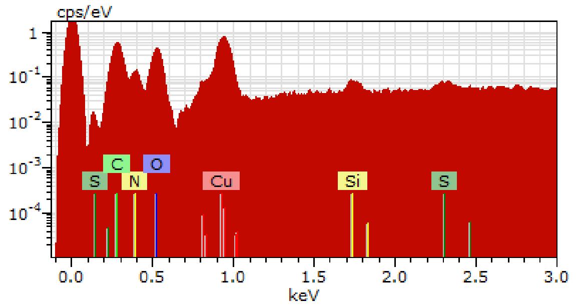

SI Figure 10. Standard HKUST-1 powder after prior exposure to H_2O and subsequent exposure to ammonia. Note the increase in the N peak at ~0.4 keV relative to SI Figure 7b.

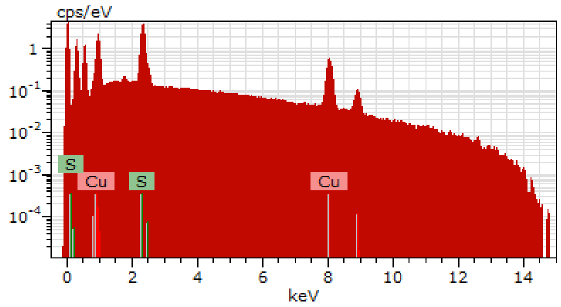

SI Figure 11. Standard HKUST-1 powder after exposure to DMSO. Quantification yielded 45% Cu and 55% S. Note that in comparison to as-received sample data in SI Figure 7a, the S peak at 2.3 keV increased significantly and is similar to as-synthesized sample data in SI Figure 1a

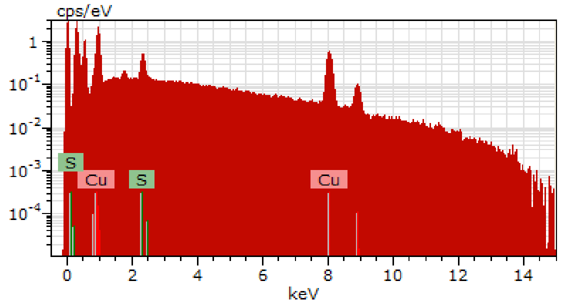

SI Figure 12. Standard HKUST-1 powder after prior exposure to DMSO and subsequent exposure to ammonia. Note that in comparison to SI Figure 11, the S peak at 2.3 keV is significantly reduced and N peak at ~0.4 keV becomes apparent. (90% Cu, 10% S)
